# Photocatalytic Water Splitting for O_2_ Production under Visible Light Irradiation Using NdVO_4_-V_2_O_5_ Hybrid Powders

**DOI:** 10.3390/ma10040331

**Published:** 2017-03-23

**Authors:** Tzu Hsuan Chiang, Tso-Ming Chen

**Affiliations:** Department of Energy Engineering, National United University, Lienda, Nan-Shi Li, Miaoli 36006, Taiwan; click042053@gmail.com

**Keywords:** glycothermal method, photocatalytic, oxygen evolution, visible light irradiation

## Abstract

The study investigated photocatalytic water splitting for O_2_ production under visible light irradiation using neodymium vanadium oxide (NdVO_4_) and vanadium oxide (V_2_O_5_) hybrid powders. The results in a sacrificial agent of 0.01 M AgNO_3_ solution were obtained, and the highest photocatalytic O_2_ evolution was 2.63 μmol/h, when the hybrid powders were prepared by mixing Nd and V at a volume ratio of 1:3 at a calcination temperature of 350 °C for 1 h. The hybrid powders were synthesized by neodymium nitrate and ammonium metavanadate using the glycothermal method in ethylene glycol at 120 °C for 1 h. The hybrid powders consisted of two shapes, NdVO_4_ nanoparticles and the cylindrical V_2_O_5_ particles, and they possessed the ability for photocatalytic oxygen (O_2_) evolution during irradiation with visible light. The band gaps and structures of the hybrid powders were analyzed using UV-visible spectroscopy and transmission electron microscopy.

## 1. Introduction

In recent years, neodymium vanadium oxide (NdVO_4_) has been studied extensively. The structure of NdVO_4_ depends on the Nd^3+^ ion, which is dodecahedrally surrounded by eight oxygen ions between the neighboring tetrahedral (VO_4_^3−^) [1], which belongs to ABO_4_-type structures. NdVO_4_ has unique properties, such as luminescence [2], magnetization [1] and as a photocatalytic degradation dye under irradiation with visible light [3] or ultraviolet light [4,5]. Oshikiri and Boero [6] reported that NdVO_4_ is used to produce hydrogen from water and an aqueous solution of methanol under a 400-W Hg high-pressure lamp using an inner-irradiation-type quartz cell. Recently, several techniques have been used for the synthesis of NdVO_4_ particles, such as the two steps of the hydrothermal reaction at 180 °C [7], the sonochemical method [1], microwave synthesis [8] and the Czochralski method at ambient pressure in a nitrogen atmosphere [9]. Solvothermal synthesis in ethylene glycol is a glycothermal method, also called the polyol method [10], which is a process with a lower synthesis temperature than the solid state, hydrothermal and flux methods. The glycothermal method usually uses ethylene glycol or other polyols as the solvent and as reducing and stabilizing agents that can control the size and shape of the particles [11]. When polyols are used as solvents, they reduce the volume fraction of the water in the nucleation reaction, which strongly facilitates the synthesis of nanocrystallites. Some reports have confirmed the significance of this method to synthesize various particles, such as Fe_3_O_4_ microspheres [12], flower-like LiMnPO_4_ nanostructures self-assembled with nanobelts [13], ZnWO_4_ nanocrystals and nanorods [14],LiFePO_4_ nanoparticles [15], CuO/CeO_2_ [16] and flower-like LiMnPO_4_ [13].

For the past few years, there have been fewer types of O_2_ evolution photocatalysts than H_2_ evolution photocatalysts for the photocatalysis of water splitting. Various materials have been used as O_2_ evolution photocatalysts under visible light irradiation, such as BiVO_4_ [14,15,16,17,18], WO_3_ [19], Y_2_MSbO_7_ (M = Ga, In, Gd) [20], Ag_3_PO_4_ [21] and Bi_2_MoO_6_ [22]. In addition, the overall water splitting is a difficult reaction to initiate by one-step photoexcitation under visible light using a photocatalyst. Generally, it can be initiated through half reactions in sacrificial reagents, such as methanol or silver nitrate (AgNO_3_), which act as hole or electron scavengers to exhibit activities independently for the evolution of H_2_ and O_2_. When a photocatalyst is used in the photocatalytic water splitting, the top of the valence band of the photocatalyst must be located at a more positive level than the oxidation potential of the water. Furthermore, the bottom of the conduction band must be located at a more negative level than the reduction potential of the water.

Xu et al. [3] reported that NdVO_4_ nanowires possess photocatalytic degradation of Rhodamine B and methyl orange, and Jiang et al. [23] reported that the V_2_O_5_/BiVO_4_-composite enhanced photocatalytic activity for the degradation of methylene blue. To date, we have found no research focused on the effect of NdVO_4_ particles on the photocatalytic O_2_ evolution during irradiation with visible light. Herein, we describe our synthesis of pure V_2_O_5_ and NdVO_4_-V_2_O_5_ hybrid powder photocatalysts using the glycothermal method, and we demonstrate the microstructure, crystal phase and the efficiency of photocatalytic O_2_ evolution using irradiation with visible light (λ > 420 nm) in an AgNO_3_ solution at different calcination temperatures of the hybrid powders.

## 2. Experimental Section

### 2.1. Preparation of the NdVO_4_-V_2_O_5_ Hybrid Powders

The ammonium metavanadate (NH_4_VO_3_, supplied by Sigma-Aldrich Co., Ltd., St. Louis, MO, USA) was not dissolved in distilled water at room temperature. It was added to 50 mL of distilled water and then heated to 80 °C for desolvation, then other distilled water prepared with a 0.1 M NH_4_VO_3_ solution was added. Various volumes (i.e., 300, 150, 100 and 75 mL) of a 0.1 M neodymium nitrate (NdNO_3_) solution were mixed with 300 mL of the 0.1 M NH_4_VO_3_ solution (as Nd/V mixed with 1:1, 1:2, 1:3, and 1:4 volume ratios) and 50 mL of ethylene glycol (provided by Showa Chemical Co., Ltd., Tokyo, Japan), respectively. When the mixture was heated at 120 °C for 1 h, a precipitate was obtained. The precipitate was washed with distilled water five times and placed in the oven at 80 °C for 24 h. The microstructures were observed using JEOL JED-2300 field emission scanning electron microscopy (SEM, JEOL Ltd., Tokyo, Japan), and the samples was analyzed using selected-area electron diffraction (SAED, FEI, Hillsboro, TX, USA). High-resolution transmission electron microscopy (HRTEM) images were obtained using a Philips TECNAI 20 microscope (FEI, Hillsboro, TX, USA). The surface areas of the hybrid powders were measured by the conventional Brunauer–Emmett–Teller (BET) method (BEL Japan, Inc., Tokyo, Japan). The X-ray diffraction (XRD) data were collected over the 2θ range of 10°–100° by a Rigaku TTRAXIII rotating anode diffractometer with a Ni-filtered, Cu-K radiation source (wavelength of 1.54 Å). UV-Vis diffuse reflectance absorption spectra (DRS) were supplied by a JASCO International Co., Ltd. (Model: JASCO V-670 spectrophotometer, Tokyo, Japan) equipped with a PIN-757 integrating sphere, where the baseline was recorded using a barium sulfate reference. The Raman spectra at room temperature were obtained with a Micro Raman Identify Spectrometer (Model: MRI532S, ProTrusTech Co., Ltd., Tainan, Taiwan) using a 532-nm laser.

### 2.2. Measurement of Photocatalytic Activity

The impregnation method was used to load all of the particles with 0.5 wt % of the co-catalyst as mixed oxides of rhodium and chromium (Rh2-yCryO_3_) [24]. In this method, the particles were prepared with 0.5 wt % of Na_3_RhCl_6_·nH_2_O (Mitsuwa Chemistry Co., Ltd., Hiratsuka-shi, Japan, Rh 17.8 wt %) and 0.5 wt % of Cr(NO_3_)_3_·9H_2_O (Kanto Chemicals Co., Inc., Tokyo, Japan, 98.0%–103.0%) as the Rh and Cr sources, respectively, and calcined in air at 350 °C for 1 h. All reactions took place in 100 mL of a 20-mM AgNO_3_ solution mixed with 200 mg of NdVO_4_ particles; the particles were kept suspended with magnetic stirring, and they were irradiated with a top-irradiation-type reactor using a 300-W Xe CERMAX lamp (λ > 420 nm). Oxygen evolution was detected with an online gas chromatograph (GC) equipped with a thermal conductivity detector (Shimadzu, GC-8A, Kyoto, Japan) using Ar at a pressure of 0.2 MPa as the carrier gas. A calibration curve was used to correlate the response generated by the TCD on the GC to the actual gas composition. By injecting known concentrations (and thus, known molar amounts) of gas and then monitoring the response, a graph of area (y) versus concentration (x) was plotted. Then, by solving the equation of a line (y = mx + c), a concentration can be calculated for any area. Therefore, the amount of O_2_ evolved that was calculated based on the calibration curve was represented by the following equation:

Y = 5.33 × 10^−5^X (1)

where Y is the area of GC detected and X is the amount of O_2_ evolved.

## 3. Results and Discussion 

### 3.1. The Formation of Hybrid Powders

The study used ethylene glycol as a solvent. In the glycothermal process, when the mixture is heated to 120 °C, the ethylene glycol undergoes oxidization, forming acetaldehyde, as shown in Equation (2) and confirmed in an earlier publication [25]. The acetaldehyde formed acetic acid, which easily was dissociated into acetate ions (CH_3_COO^−^) and hydrogen ions (H^+^), as shown in Equation (3). In addition, the NdNO_3_ and NH_4_VO_3_ were dissociated to neodymium ions (Nd^+^), as shown in Equation (4), and vanadium ions (VO_3_^−^), as shown in Equation (5). The Nd^+^ reacted with VO_3_^−^ and H^+^ (from Equation (3)) and then was oxidized to form neodymium vanadium oxide hydrate (NdVO_4_·H_2_O), as in Equation (6); some of the VO_3_^−^ ions formed vanadium pentoxide hydrate (V_2_O_5_·H_2_O), as shown in Equation (7). The hybrid powders were dried at 60 °C for 24 h, and the water was eliminated, forming NdVO_4_ and V_2_O_5_ compounds.

2CH_2_OHCH_2_OH + O_2_ → 2CH_3_CHO + 2H_2_O (2)

2CH_3_CHO + O_2_ → 2CH_3_COOH → CH_3_COO^−^ + H^+^ (3)

NdNO_3_ → Nd^+^ + NO_3_^−^ (4)

NH_4_VO_3_ → VO_3_^−^+ NH_4_^+^ (5)

Nd^+^ + VO_3_^−^ + 2H^+^ + O_2_ →NdVO_4_·H_2_O (6)

2VO_3_^−^ + 2H^+^ → V_2_O_5_·H_2_O (7)

**Figure 1:**
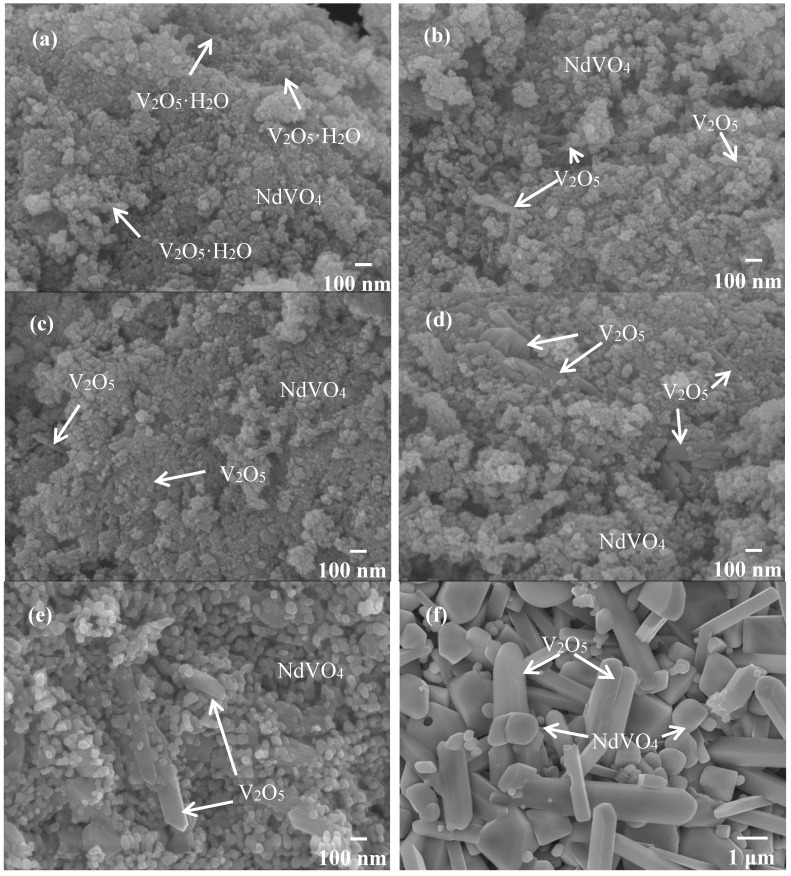
SEM images of the hybrid powders prepared with Nd/V mixed at the 1:2 volume ratio under various calcination temperatures: (**a**) without calcination; (**b**) at 200 °C; (**c**) at 300 °C; (**d**) at 400 °C; (**e**) at 500 °C; (**f**) at 600 °C.

**Figure 2:**
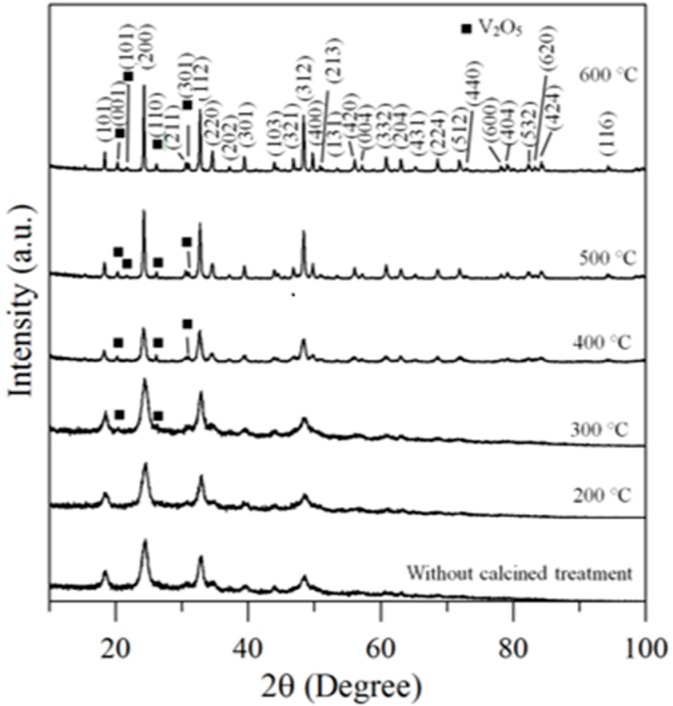
XRD data for hybrid powders prepared at Nd/V mixed at the 1:2 volume ratio at various calcination temperatures for 1 h.

Therefore, the hybrid powders, NdVO_4_ and V_2_O_5_ particles existed simultaneously in the precipitate as demonstrated in the SEM images shown in Figure 1a. However, only the peaks of the NdVO_4_ nanoparticles were obtained in the XRD data, as shown in Figure 2, and the V_2_O_5_ rods may have a very low volumetric content that might have been below the detection limit of the XRD diffractometer. When the hybrid powders were subjected to calcination temperature at 300 °C, V_2_O_5_ peaks began to appear. In addition, the width of the cylindrical particles obviously increased when the calcination temperature was more than 400 °C, as shown in Figure 1d–f. The NdVO_4_ and V_2_O_5_ compounds both have crystallization structures that are dependent on more crystalline peaks appearing when the calcination temperature is greater than 400 °C, as shown in the XRD data in Figure 2. All of the peaks can be indexed for the tetragonal NdVO_4_ structure a = 7.335 Å, c = 6.431 Å, which is in good agreement with the values presented in the literature (JCPDS 15-0769) with the space group as *I*4_1_/amd [8]. Regardless, this means that it crystallizes in the tetragonal structure composed of a slightly-distorted VO_4_^3^^−^ tetrahedral and the rare earth ion, Nd^3+^, between the neighboring tetrahedral [5]. In addition, the peaks (black squares) at the 2θ values of 20.1° and 21.5° match the orthorhombic V_2_O_5_ structure (JCPDS 41-426), suggesting that it is probable that some of the elemental vanadium is produced by being segregated from the hybrid powders. The results confirmed that the hybrid powders consisted of two structures, i.e., NdVO_4_ and V_2_O_5_.

**Figure 3:**
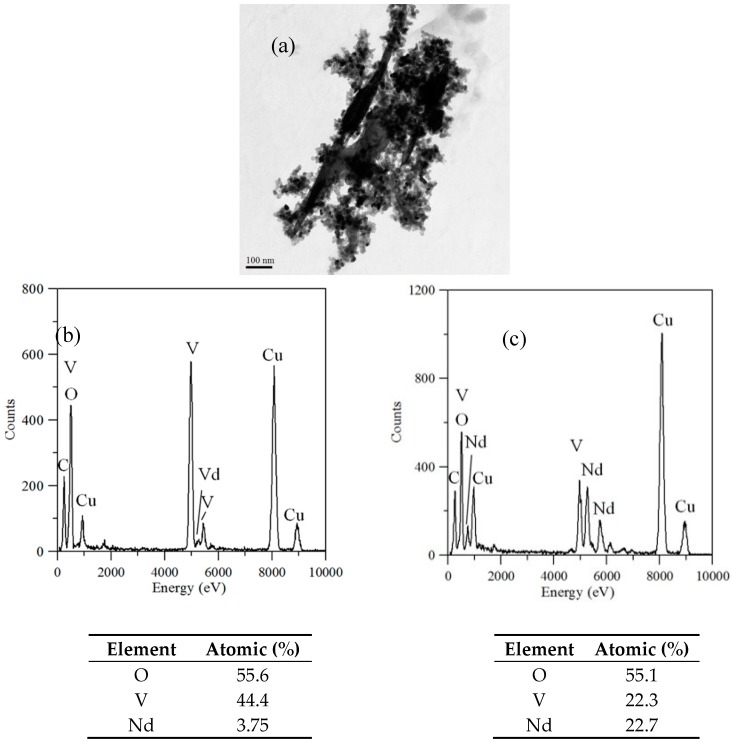
(**a**) TEM image; (**b**) TEM-EDX of a cylindrical particle; (**c**) TEM-EDX nanoparticle of hybrid powders at a calcination temperature of 400 °C for 1 h.

**Figure 4:**
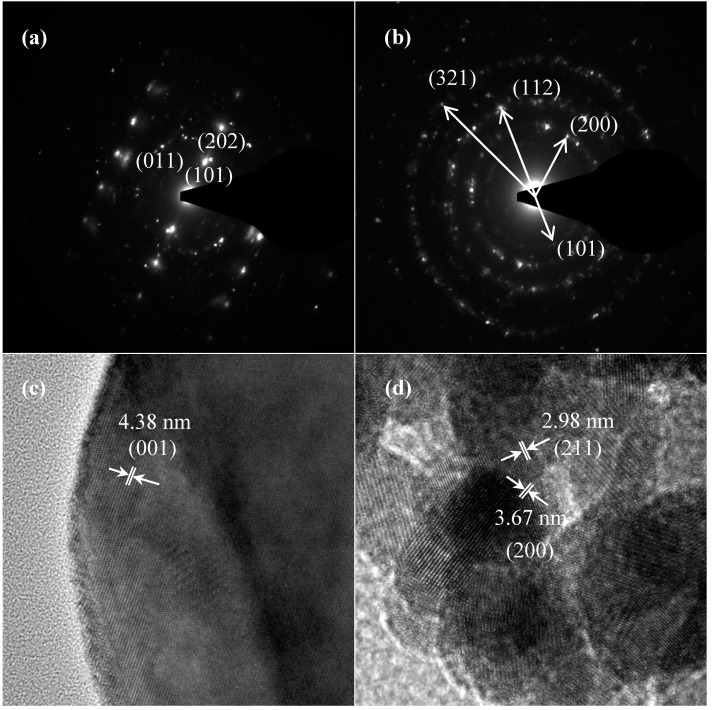
SAED patterns: (**a**) cylindrical particles; (**b**) nanoparticles; HRTEM of: (**c**) cylindrical particles; (**d**) nanoparticles of NdVO_4_-V_2_O_5_ hybrid powders at the calcination temperature of 400 °C for 1 h.

TEM and TEM-EDX analyses were used to distinguish the nanoparticle and cylindrical particle components of the hybrid powders, respectively, as shown in Figure 3a–c. Figure 3b shows that the cylindrical particles consisted of 3.78% Nd elements and 44.4% of V elements, making the V_2_O_5_ structure possible. Figure 3c shows that the stoichiometric atomic ratio of Nd/V was one for the nanoparticles that possibly belonged to the stoichiometric NdVO_4_. In addition, the structures of the two-shaped particles were confirmed by the SAED patterns and HRTEM images. The (101), (202) and (011) of the SAED patterns shown in Figure 4a and the lattice fringes of 4.38 nm corresponding to (001) of HRTEM in Figure 4b confirmed the V_2_O_5_ structure. The (101), (200), (112) and (321) of the SAED patterns in Figure 4c and the lattice fringes of about 0.367 and 0.298 nm corresponding, respectively, to the (200) and (211) planes of the NdVO_4_ structure in Figure 4d also were identified.

### 3.2. Photocatalytic Activity of Hybrid Powders on O_2_ Evolution

**Figure 5:**
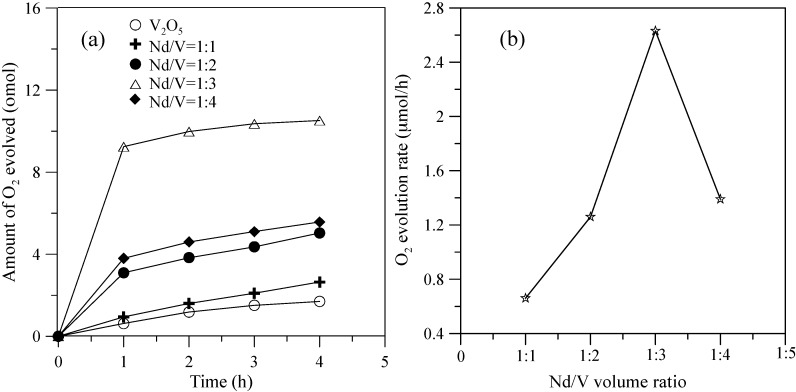
(**a**) Photocatalytic O_2_ evolution; (**b**) O_2_ evolution rate of the hybrid powders based on the Nd/V volume ratio during irradiation with visible light for 4 h.

Figure 5 shows the hybrid powders’ photocatalytic O_2_ evolution using various Nd/V volume ratios from an aqueous AgNO_3_ solution subjected to visible light irradiation (λ > 420 nm). In every case, the slope of the photocatalytic O_2_ evolution was greater in the first hour than at any time after that due to the degradation of the activity of the photocatalysts, which resulted from the photodeposition of Ag on the surfaces of the powders. The results indicated that the maximum activity of the hybrid powders synthesized by the Nd/V mixture at a 1:3 volume ratio was 2.63 μmol/h, as shown in Figure 5. The pure V_2_O_5_ powders had the lowest photocatalytic O_2_ evolution. However, the distinctive absorption edges of the pure V_2_O_5_ powders at 620 nm were demonstrated by DSR, and the band gap was 2.02 eV. The direct band gap of 2.3 eV and an indirect gap of 1.9 eV were obtained using Equation (7) [26] when the value of m changes during the hypothesized direct-allowed transition (m = 1/2) and indirect-allowed transition (m = 2). The results were the same as those reported by Chakrabarti et al. [27].

α = α_0_(hv − E_g_)m (8)

where α is the absorption coefficient, α_0_ is a constant, hv is the energy of a photon and E_g_ is the band gap.

**Figure 6:**
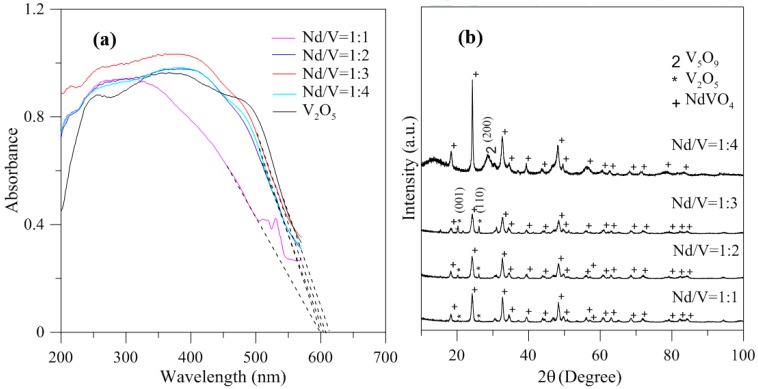
(**a**) DRS of hybrid powders with different Nd/V volume ratios; (**b**) XRD of hybrid powders with different Nd/V volume ratios.

**Figure 7:**
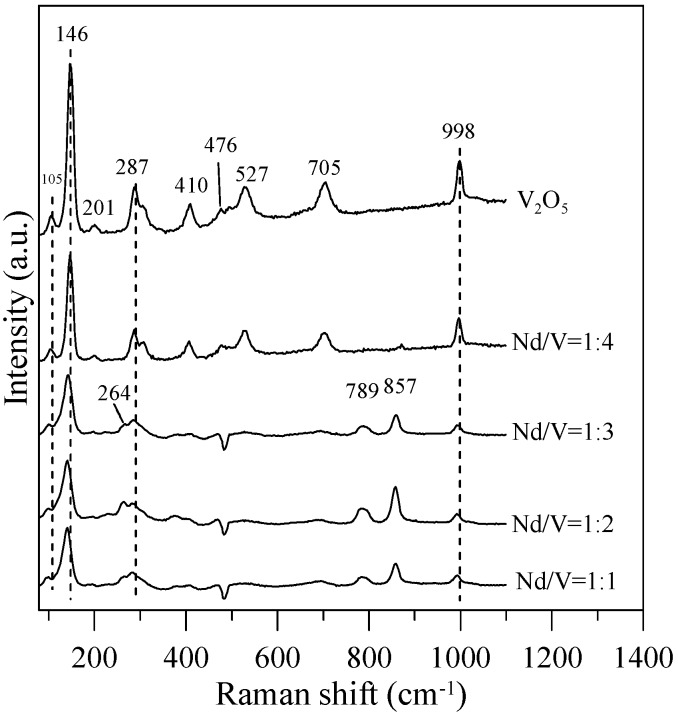
Raman spectra of the hybrid powders for different Nd/V volume ratios.

In addition, the results indicated that the hybrid particles prepared by different Nd/V mixtures at 1:1, 1:2, 1:3 and 1:4 volume ratios obtained distinctive absorption edges at 590, 605, 615 and 605 nm, respectively, and its energy gaps were estimated to be 2.10, 2.05, 2.02 and 2.05 eV, respectively, according to the intercept on the wavelength axis for a tangent line, as shown in Figure 6. The results showed that the hybrid powders, irrespective of whether they had direct band gaps, indirect band gaps or energy gaps, followed the same results as Nd/V mixed at 1:3 < 1:4 = 1:2 < 1:1 volume ratios, as shown in Table 1. The direct band gaps of all of the powders were lower than the direct band gap of NdVO_4_, which was 2.86 eV [8]. The direct band gap of V_2_O_5_, i.e., 2.3 eV, caused the absorption edges to shift to positive wavelengths if hybrid powders had increased contents of V_2_O_5_ compounds. However, Nd/V was mixed at a 1:4 volume ratio; it had a higher band gap than it had at the 1:3 volume ratio. As shown in Figure 6a, the wavelength was shifted to 605 nm due to the vanadium dioxide (V_5_O_9_) structure that formed, as confirmed by the plane (200) of peak, as shown in Figure 6b where the triangle symbol appears (JCPDS 80-2027) and by the disappearance of planes (001) and (110) of the peaks on the V_2_O_5_ structure. In addition, the results of the study indicated that the Raman bands of the V_2_O_5_ powders corresponded to those reported by Sanchez et al. [28] and Bhat et al. [29], as shown in Figure 7. The Raman bands of the Nd/V = 1:4 powders were similar to those of the V_2_O_5_ powders. This reason was that the photocatalytic O_2_ evolution of the Nd/V = 1:4 was less than that of Nd/V = 1:3.

In Figure 7, the Raman bands of 264, 789,and 857 cm^−1^ can be assigned to the symmetric bending vibrations of VO_4_^3−^ anions, the anti-symmetric stretching vibrations of VO_4_^3−^ and the symmetric VO_4_^3−^ stretching mode for Nd/V = 1:1, 1:2 and 1:3 powders, respectively. Thalluri et al. [30] reported that the variations in one of the V-O bond lengths among the powders can be explained on the basis of the packing of the structure. The stronger the packing formed, the shorter the V-O bond length is and the higher the photocatalytic activity is. In comparison with the Raman bands of the pure V_2_O_5_ structure for Nd/V = 1:1, 1:2 and 1:3 powders, the bands shifted 105, 146, 287 and 998 cm^−1^ toward the higher wavenumber. From the Raman shift position that corresponds to the symmetric stretching mode, it is possible to attain information about the length of the V-O bonds in the VO_4_^3−^ tetrahedron by using the following expression as the Raman wavelength, i.e., ν cm^−1^ = 21,349 exp(−1.9176 *R*) [31,32]. The results indicate that the lengths (R) of the V-O bonds in the Nd:V = 1:3 powders are shorter than those of the other two hybrid powders, which demonstrates that the Nd:V = 1:3 powders have a greater photocatalytic O_2_ evolution. From the results above, it is obvious that greater contents of V_2_O_5_ compounds improve the photocatalytic activity of hybrid powders. 

**Figure 8:**
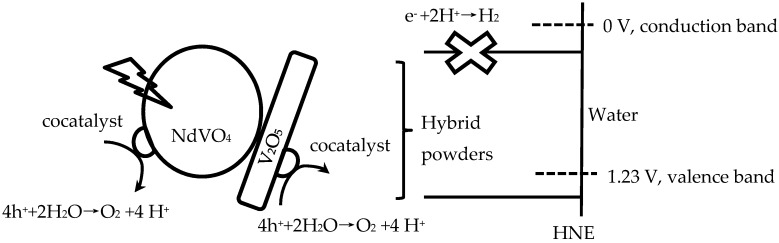
Schematic of hybrid powders for photocatalytic O_2_ production.

Therefore, NdVO_4_-V_2_O_5_ hybrid powders adsorb light and generate more electrons and holes, then charge separation and migration to the surfaces of the NdVO_4_-V_2_O_5_ hybrid powders, and the valence band is below the water oxidation potential (1.23 V versus NHE at pH 0). The results indicated that the holes of NdVO_4_-V_2_O_5_ hybrid powders can oxidize water molecules to form oxygen, as shown in Figure 8. However, the conduction of the hybrid powders perhaps was lower than the water reduction potential of 0 V, which caused the electron of the hybrid powders to be unable to reduce H+ to form hydrogen in water or in a sacrificial agent solution (10% methanol). Based on the stoichiometry of NdVO_4_, the oxidation states and atomic orbitals are given by Nd^3+^ (6s2,4f4), V^5+^ (3d0) and O_2_^−^ (2p6). The valence band is formed by coupling the Nd 6s and O 2p orbitals, while the conduction band is controlled primarily by V 3d orbitals, with contributions from the O 2p and Nd 4f orbitals.

**Figure 9:**
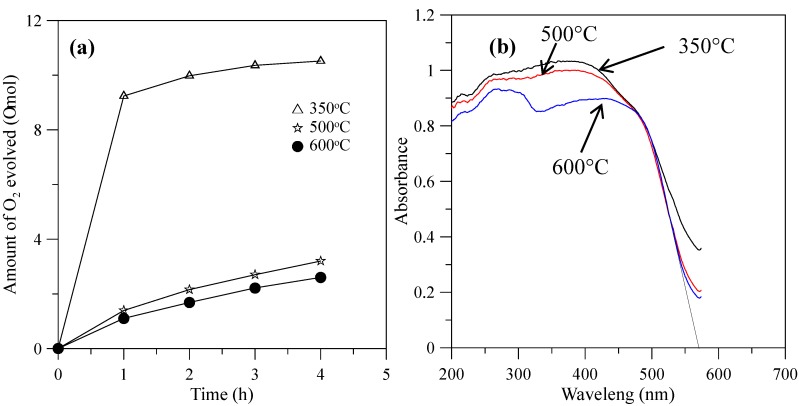
(**a**) Photocatalytic O_2_ evolution; (**b**) DRS of the hybrid powders prepared by Nd/V mixed at a 1:3 volume ratio at calcination temperatures of 350–600 °C.

**Figure 10:**
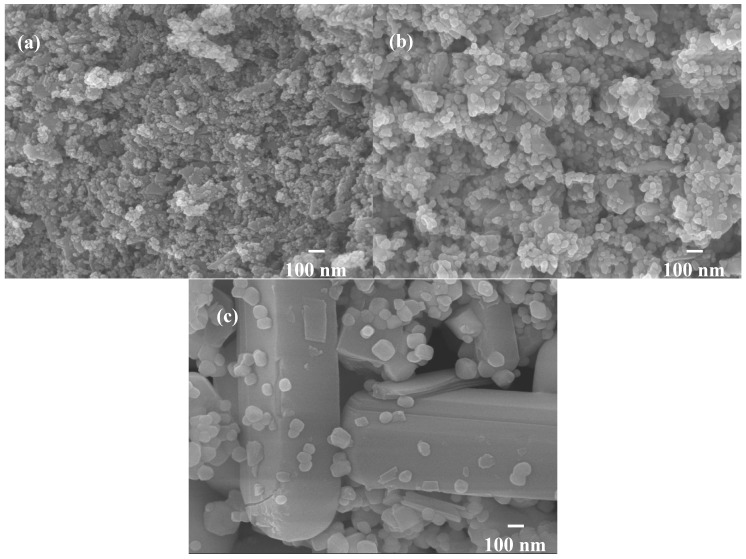
SEM images of hybrid powders prepared by Nd/V mixed at the 1:3 volume ratio at calcination temperatures of: (**a**) 350 °C; (**b**) 500 °C; (**b**) 600 °C.

**Figure 11:**
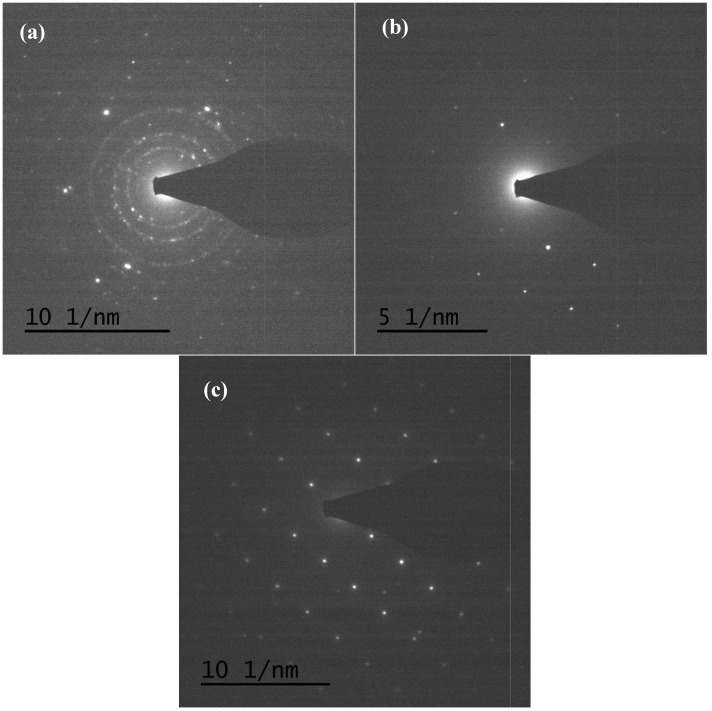
TEM images of (V_2_O_5_) hybrid powders prepared by Nd/V mixed at the 1:3 volume ratio at calcination temperatures of: (**a**) 350 °C; (**b**) 500 °C; (**c**) 600 °C.

When the hybrid powders were calcined in the temperature range from 350–600 °C, the photocatalytic activity of O_2_ evolution decreased, as shown in Figure 9a. The extent of the decrease depended on the increase in the particle size of the powders, as shown in Figure 10, and their surface areas decreased, as shown in Table 2. The hybrid powders were calcined at 500 °C and 600 °C, for which their absorption edges at 570 nm were smaller than 350 °C at 615 nm, as shown in Figure 9b. Therefore, they had lower photocatalytic activity for O_2_ evolution. Jafari et al. [33] reported that high crystallinity and a small particle size of the powders are desired to minimize the photo-generated electron and hole recombination for water splitting, and the higher crystallinity of the particles was concomitant with the formation of larger particles when the particles are subjected to higher calcination temperatures. The hybrid powders used in this study produced the same results, which were demonstrated by crystallite size using XRD (Table 2) and the TEM images in Figure 11 when the hybrid powders were calcined at 350–600 °C. The crystallinity and particle sizes increased as the calcination temperature increased, and the surface area and the photocatalytic activity of O_2_ evolution decreased as the calcination temperature increased. These results indicated that smaller particle sizes (i.e., larger surface areas) of the hybrid powders were more effective for O_2_ evolution than higher crystallinity of the hybrid powders. Pai et al. [34] reported that optimum surface area and crystallinity are required for suitable performance of the photocatalyst. Because the large surface area of the photocatalyst increased as the amount of water molecules adsorbed increased, the reaction of the photo-excited electron holes with the substrate was enhanced. Additionally, the large surface area generated defect centers in the photocatalyst, because the surface of a catalyst where the continuity of a crystal terminates can be considered a defective site. These defective sites are assumed to be the electron-hole recombination centers. Therefore, the larger the surface area, the faster the reaction, and the smaller the surface area, the lesser the electron-hole recombination becomes.

## 4. Conclusions

The NdVO_4_-V_2_O_5_ hybrid powders were synthesized successfully via a glycothermal method in ethylene glycol. The band gap energies of the hybrid powders were obtained between 2.02 and 2.60 eV, and the valence bands of the NdVO_4_-V_2_O_5_ hybrid powders were below the water oxidation potential. Therefore, they had photocatalytic activity resulting in O_2_ evolution. The greatest rate of photocatalytic O_2_ evolution, 2.63 μmol/h, was obtained using visible light irradiation when the Nd/V was mixed at a 1:3 volume ratio and calcined at the temperature of 350 °C for 1 h. The results of the study demonstrated that hybrid powders treated at high calcination temperatures have high crystallinity, which impaired their photocatalytic activity and O_2_ evolution. Because they had larger particle sizes, they had smaller surface areas, and this resulted in their absorbing fewer water molecules, which decreased their photocatalytic activity. The results of the study indicated that the smaller particle sizes of the powders were more effective for inducing O_2_ evolution than their higher crystallinities.

## Figures and Tables

**Table 1:** Band gaps and energy gaps of hybrid powders for different Nd/V volume ratios.

Nd/V Volume Ratio	Direct Band Gap (eV)	Indirect Band Bap (eV)	Energy Gap (eV)
1:1	2.2	2.73	2.10
1:2	2.16	2.65	2.05
1:3	2.14	2.60	2.02
1:4	2.17	2.65	2.05

**Table 2:** Physical properties and O_2_ evolution activities of BiVO_4_ samples synthesized at different calcination temperatures.

Sample	Crystallite Size (nm)	BET Surface Area (m^2^/g)	O_2_ Evolution Rate (μmol/h)
350 °C	13.5	92.84	2.63
500 °C	36.7	75.35	0.80
600 °C	62.9	48.67	0.65
